# MosqTent: An individual portable protective double-chamber mosquito trap for anthropophilic mosquitoes

**DOI:** 10.1371/journal.pntd.0005245

**Published:** 2017-03-09

**Authors:** José Bento Pereira Lima, Allan Kardec Ribeiro Galardo, Leonardo Soares Bastos, Arthur Weiss da Silva Lima, Maria Goreti Rosa-Freitas

**Affiliations:** 1 Laboratório de Fisiologia e Controle de Artrópodes Vetores, Instituto Oswaldo Cruz, Fiocruz, Rio de Janeiro, Brazil; 2 Laboratório de Entomologia, Instituto de Biologia do Exército, Rio de Janeiro, Brazil; 3 Instituto de Pesquisas Científicas e Tecnológicas do Estado do Amapá, Macapá, Brazil; 4 Programa de Computação Científica, Fiocruz, Rio de Janeiro, Brazil; 5 Laboratório de Mosquitos Transmissores de Hematozoários, Instituto Oswaldo Cruz, Fiocruz, Rio de Janeiro, Brazil; Mahidol University, THAILAND

## Abstract

Here, we describe the development of the MosqTent, an innovative double-chamber mosquito trap in which a human being attracts mosquitoes while is protected from being bitten within the inner chamber of the trap, while mosquitoes are lured to enter an outer chamber where they are trapped. The MosqTent previously collected an average of 3,000 anophelines/man-hour compared to 240 anophelines/man-hour for the human landing catch (HLC), thereby providing high numbers of human host–seeking mosquitoes while protecting the collector from mosquito bites. The MosqTent performed well by collecting a high number of specimens of *Anopheles* *marajoara*, a local vector and anthropophilic mosquito species present in high density, but not so well in collecting *An*. *darlingi*, an anthropophilic mosquito species considered the main vector in Brazil but is present in low-density conditions in the area. The HLC showed a higher efficiency in collecting *An*. *darlingi* in these low-density conditions. The MosqTent is light (<1 kg), portable (comes as a bag with two handles), flexible (can be used with other attractants), adaptable (can be deployed in a variety of environmental settings and weather conditions), and it can be used in the intra-, peri-, and in the extradomicile. Also, the MosqTent collected similar portions of parous females and anthropophilic mosquito species and collects specimens suitable for downstream analysis. Further developments may include testing for other fabric colors, different mesh sizes and dimensions for other hematophagous insects and conditions, additional chemical mosquito attractants, and even the replacement of the human attractant in favor of other attractants. MosqTent modifications that would allow the trap to be applied as a vector control tool with killing action could also be explored.

## Overview

### Introduction

Given the preference of vector-borne human parasites to feed on human blood, most adult mosquito traps currently in use are far less efficient than human landing catches (HLCs) for collecting host-seeking mosquitoes. A clear picture of the dynamics and transmission risks posed by anthropophilic mosquitoes in disease-endemic areas is provided by the use of HLC. HLC is logistically complex, and ethical dilemmas arise when fieldworkers may be exposed to the risk of infective biting and acquiring deadly or unknown pathogens. To address the problem of how to attract and assess anthropophilic mosquitoes while protecting the human bait from being bitten, we have designed and tested a novel, portable, double-chamber mosquito trap, the MosqTent.

### Methods

The MosqTent consists of an isolated inner chamber and an outer chamber to collect mosquitoes that are attracted by human cues (visual, temperature, and odor, among other cues—hereafter referred to as "human-attractant"), which is protected within the inner chamber. Two versions of the MosqTent (black versus white) were tested in the Brazilian Amazon against the Biogents (BG)-sentinel trap + CO_2_, the protected HLC (human attractant wearing a protective sock), and the HLC. The HLC was considered the gold standard for data analysis.

### Results

The MosqTent collected all anopheline species caught by the HLC. The MosqTent collected more mosquitoes than the HLC in high mosquito–density conditions (3,000 anophelines/man-hour compared to only 240 anophelines for the HLC). The MosqTent performed well by collecting a high number of *Anopheles marajoara* specimens, a local anthropophilic vector present in high density, but not so well in collecting *An*. *darlingi*, an anthropophilic species that is considered the main Brazilian vector but is present in low-density conditions in the area. The HLC showed higher efficiency in collecting *An*. *darlingi* in these low-density conditions. For *An*. *marajoara*, the MosqTent white and black collected nearly twice as many specimens as the HLC (25.7% and 28.9%, respectively; HLC: 15.1%). For *An*. *triannulatus*, the BG compared to the HLC and tent traps were much lower (BG: 33.4%; MosqTent white and black: 10.1% and 6.4%, respectively). For *An*. *darlingi*, both tent traps caught half as many as the HLC (MosqTent white and black: 12.5% and 14.9%, respectively; HLC: 35.5%). For *An*. *braziliensis*, there was no significant difference between the MosqTent white (20.2%) and the HLC (17.6%). For *An*. *nuneztovari*, the MosqTent white collected twice as much as the HLC (MosqTent white: 34.7%; HLC: 15.5%), while the MosqTent black collected the same amount as the HLC (15.5%). The MosqTent collected *An*. *darlingi* and *An*. *marajoara* with the same parity proportions as the HLC (*An*. *darlingi*—HLC: 58.3%, MosqTent white: 59.6%, *X*^*2*^ = 3.55, df = 4, *p* = 0.47; *An*. *marajoara*—HLC: 50.4%, MosqTent white: 50.8%, *X*^*2*^ = 3.03, df = 4, *p* = 0.5).

### Discussion

The high efficiency of the MosqTent is probably due to the high mosquito density presented by *An*. *marajoara*. In these conditions, many mosquitoes bite and escape the HLC collector, who is heavily attacked and cannot collect efficiently. Possibly, this is one of the best qualities of the MosqTent. In high mosquito–density conditions, field workers are more prone to get infected in malarigenous areas.

### Conclusions

The MosqTent produces high data output by collecting large numbers of human host–seeking mosquitoes in high-density conditions while protecting the human attractant/mosquito collector from being bitten. Besides anthropophily and population density, other dynamic biological and ecological factors might influence the collection efficiency of the MosqTent.

## Background

Although malaria has decreased dramatically over the last decade, particularly in sub-Saharan Africa [1], in Brazil there were approximately 140,000 cases in 2015 [2], and the highly endemic situation persists in other countries of the Americas and Africa and in the Southeastern Asia, Western Pacific, and Eastern Mediterranean regions, with millions of cases yearly [3].

Monitoring mosquito vector populations is among the key elements of vector management strategies and assessment of mosquito-borne disease risk [4–9].

Adult mosquitoes can usually be sampled by traps, such as the CDC light-trap, the BG-sentinel, and many other models that make use of light, CO_2_, and chemical attractants [10–13] to lure mosquitoes. To transmit parasites and pathogens to humans, mosquitoes must be anthropophilic to some degree. Nonetheless, for highly anthropophilic mosquitoes, none of the existing traps are as effective as the Human Landing Catch (HLC), which uses the human catcher as an attractant. Therefore, HLCs continue to be the gold standard in some areas for vector surveillance of anthropophilic mosquito populations. Because of the importance of the HLC as a surveillance tool, even when using established guidelines [14–15], researchers and fieldwork professionals continue exposing themselves to infective bites of known and unknown pathogens.

In order to attract anthropophilic mosquitoes, while still protecting the human bait from being bitten, we present an innovative mosquito trap, the MosqTent. This trap is a double-chamber portable model for use in entomological research and by entomology surveillance teams. We present results from experiments with Neotropical malaria vectors, comparing trap data from the MosqTent against three alternative sampling tools.

## Methods

### Test site description

The MosqTent, in its final design, was tested in field conditions for anthropophilic anopheline mosquitoes in a malaria-endemic area. The area chosen was in the Northern Brazilian Amazon inside a farm located at the edge of the Ramal da Viúva road, 14 km from Cariobal, Macapá, Amapá Sate, Brazil (00° 08. 023'N, 051° 11. 131 W).

### Mosquito trap testing

**Fig 1:**
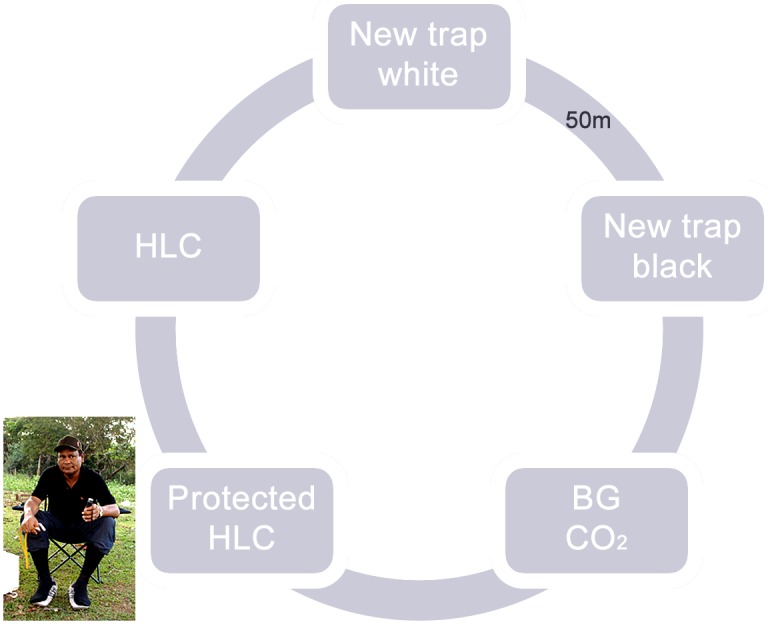
Distribution of collection points, 50 m from each other.

The new mosquito trap was tested against: a BG-sentinel trap using only CO_2_ as an attractant (CO_2_ cylinder, BioGents GmbH, Regensburg, Germany), a protected HLC where a human individual used a thick sock on one leg to increase protection from biting [10], and the traditional HLC as the gold standard. Because black is reported to increase attraction with other traps [16], and black also allows better visualization and easier collection of mosquitoes, particularly anophelines at night, we also tested a MosqTent constructed in black nylon and another in white nylon. Five distinct sites, 50 m apart from each other, were chosen for the field trials on a farm in the city of Cariobal (Fig 1).

Comparisons were carried out in a 5 x 5 Latin square design, testing each of the five sampling methods at five sites, on five consecutive nights, rotating treatments. The Latin square was repeated four times; two collections were performed in August and October 2009, which corresponds to summer, and two more collections were performed in winter, in January and April 2010.

The initial distribution of the collection methods at the five collection sites was random and was rotated clockwise during the five-night consecutive collections so that each trap was tested in each of the five collection sites (at each site only once) at a distance of 50 m from each other distributed around the farm house, where five individuals lived (Table 1).

**Table 1:** Latin square rotation scheme adopted for testing a new mosquito trap design against other collection methods for anthropophilic anophelines in Cariobal, Amapá State, Brazil.

**August 2009 Rotation scheme**					
Days	point 1	point 2	point 3	point 4	point 5
1	New trap black	New trap white	protected HLC	HLC	BGs + CO_2_
2	BGs + CO_2_	New trap black	New trap white	protected HLC	HLC
3	HLC	BGs + CO_2_	New trap black	New trap white	protected HLC
4	protected HLC	HLC	BGs + CO2	New trap black	New trap white
5	New trap white	protected HLC	HLC	BGs + CO2	New trap black
**October 2009 Rotation scheme**					
Days	point 1	point 2	point 3	point 4	point 5
1	New trap black	New trap white	protected HLC	HLC	BGs + CO2
2	BGs + CO2	New trap black	New trap white	protected HLC	HLC
3	HLC	BGs + CO2	New trap black	New trap white	protected HLC
4	protected HLC	HLC	BGs + CO2	New trap black	New trap white
5	New trap white	protected HLC	HLC	BGs + CO2	New trap black
**January 2010 Rotation scheme**					
Days	point 1	point 2	point 3	point 4	point 5
1	New trap black	New trap white	protected HLC	HLC	BGs + CO2
2	BGs + CO2	New trap black	New trap white	protected HLC	HLC
3	HLC	BGs + CO2	New trap black	New trap white	protected HLC
4	protected HLC	HLC	BGs + CO2	New trap black	New trap white
5	New trap white	protected HLC	HLC	BGs + CO2	New trap black
**April 2010 Rotation scheme**					
Days	point 1	point 2	point 3	point 4	point 5
1	New trap black	New trap white	protected HLC	HLC	BGs + CO2
2	BGs + CO2	New trap black	New trap white	protected HLC	HLC
3	HLC	BGs + CO2	New trap black	New trap white	protected HLC
4	protected HLC	HLC	BGs + CO2	New trap black	New trap white
5	New trap white	protected HLC	HLC	BGs + CO2	New trap black

Anopheline collections started half an hour before dusk and lasted for 4 h, approximately from 1800h to 2200h. For the MosqTent, the same human individual sitting as attractant inside the inner chamber in the new mosquito trap acted as the collector. After a period of 40 min sitting in the inner chamber of the trap as the attractant, the collector would leave the inner chamber fully dressed (No-see-um mesh bug jackets, pants, and hat [Bioquip, California, USA] and latex gloves) to avoid mosquito biting and captured anophelines from the outer chamber using a tube collector and transferred them to cardboard canisters in the remaining 20 min each hour. This protocol was followed by all five collectors so that yields could be compared.

### Mosquito processing

Mosquitoes captured by each collection method were separated into cardboard canisters with nylon covers, identified by type of collection method and time of collection. During identification, mosquitoes collected by the MosqTent were verified if engorged with blood. The canisters were packed in styrofoam boxes containing a moistened cloth or paper towel to maintain humidity and transported to the laboratory for identification [17–18]. A solution of 10% sucrose embedded in a piece of cotton was placed on the canister´s nylon cover. In the laboratory, mosquitoes were identified to species, and the ovaries of at least 10% of the females were dissected and examined for parity (presence of coiled tracheolar skeins) [19].

Parity was checked for the malaria vectors *An*. *darlingi* Root, 1926 and *An*. *marajoara* (Galvão and Damasceno 1942). Parity is an important entomological parameter for the life span of an anopheline species, as the older females indicate a population that has lived long enough to complete the malaria parasite cycle.

### Data analysis

Differences in the number of specimens collected for each mosquito species among the collection methods were modeled using a multilevel Poisson regression [20], controlled by collection point, month, day, and exposure hour. Collection site and month were treated as independent random effects, whereas we have included time-dependence in the random effects for day and hour, using first-order random walk. The Bayesian approach was used with vague priors, and the posterior marginals were obtained by the integrated nested Laplace approximations approach [21]. Statistical analyses were done in R 3.1.2, using the integrated nested Laplace approximations (INLA) package [22]. The posterior median and the 95% credible intervals of the capture rates for each trap were estimated and reported. Random effects for precision for the models were adjusted by mosquito species.

Differences in the number of parous females were checked by chi-square tests, adjusting the expected values by the total number of mosquitoes dissected for each collection method. All statistical analyses were carried out for *An*. *darlingi*, *An*. *marajoara*, and *An*. *triannulatus* (Neiva and Pinto 1922), the three most abundant species in the area, and for the total number of anophelines captured. Statistical analyses were done in R [22].

### Mosquito trap design

We have developed a mosquito trap named the MosqTent (patent deposited BR 10 2016 010859 4). MosqTent is a portable, double-chamber individual trap measuring 2m x 2m x 2m (height, length, and width), made in 2mm mesh white nylon, weighing <1 kg. MosqTent can be attached by strings into a gazebo-like tent (weighing <5.5 kg; S1 Supporting Information, Figs 2A, 2B, 3A and 3B) or in tree branches when it is mounted to form a cube.

**Fig 2:**
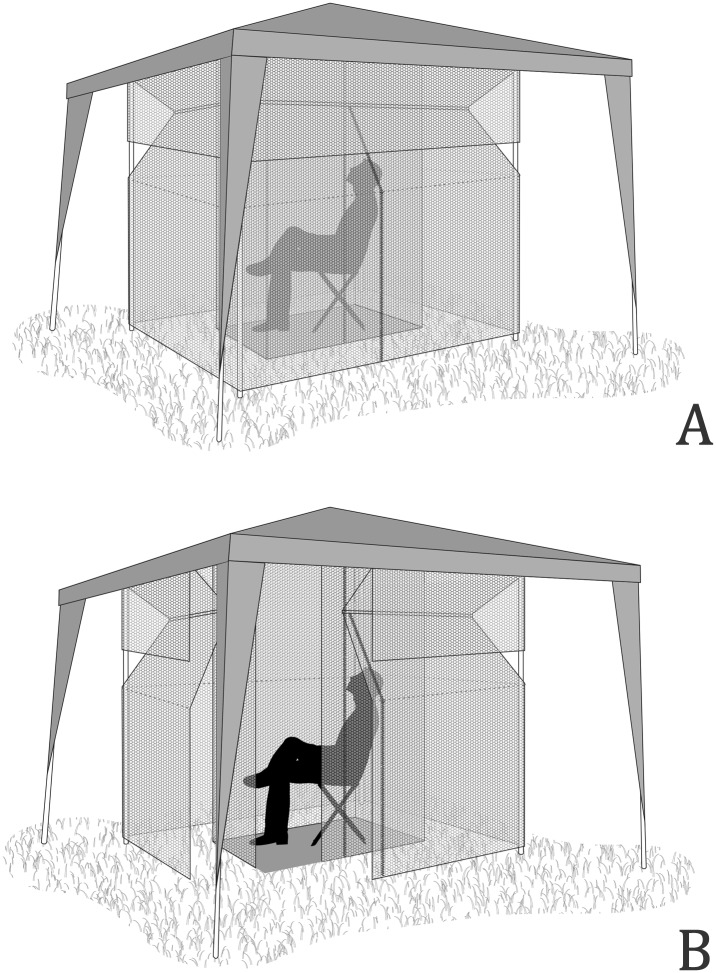
The MosqTent, a portable, double-chamber individual trap, designed to avoid mosquito biting while collecting anthropophilic mosquitoes for research and surveillance; A-outer chamber closed, B-outer and inner chamber open.

**Fig 3:**
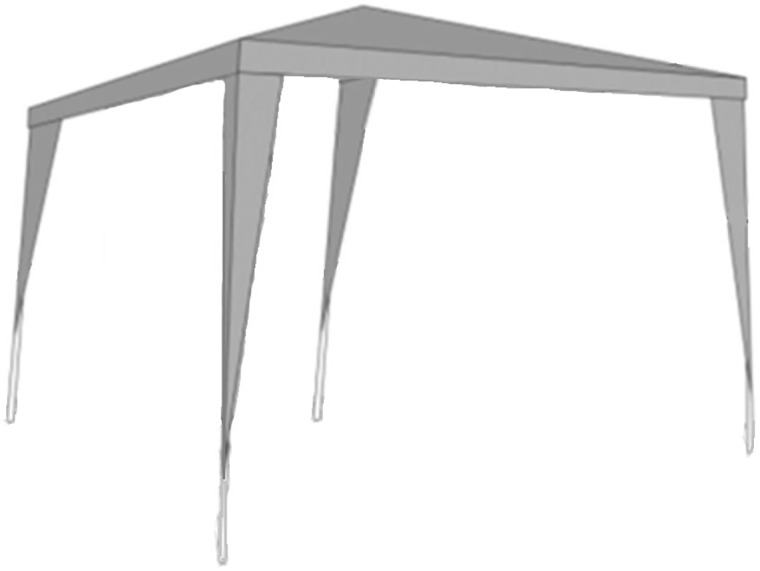
Gazebo-like tent that allows the MosqTent to be used in light rainy conditions.

The basic MosqTent design consists of an inner chamber, in which the human attractant sits throughout the sampling period (as traditionally performed for the HLCs), surrounded by an outer chamber, where the mosquitoes are trapped. The gazebo-like tent acts as an impermeable roof for protection from light rain.

**Fig 4:**
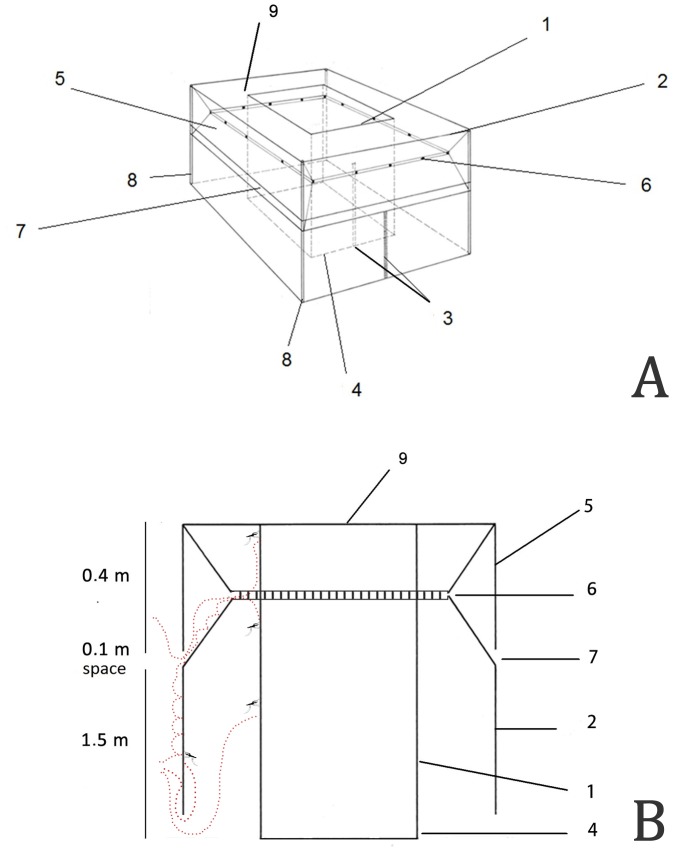
MosqTent parts; A in perspective; B side view: 1: Inner chamber, 2: Outer chamber, 3: Zippers, 4: Floor of the inner chamber, 5: Wing, 6: Holes in the wall of the outer chamber, 7: Space between the wing and the outer wall, 8: Corners, 9: Roof common to the inner and outer chambers, red dotted-lines: Alternative ways for mosquito trapping.

Mosquitoes enter the trap through spaces at the bottom of the outer chamber, where the tent material is lifted 10 cm from the ground. They are trapped when they enter the outer chamber. It was observed during the development of the trap that once the mosquitoes entered the outer chamber at ground level, they tended to fly to the top of the walls inside the outer chamber. Because of this observation, a surrounding wing, consisting of a trapezoid cut and a longitudinal row of holes was created in the wall of the outer chamber to increase collection efficiency. This design also makes it difficult for mosquitoes to escape (Fig 4).

Inside the inner chamber of the MosqTent, a human individual remains seated acting as an attractant. This inner chamber is a cube formed by a floor, a roof, and four walls completely closed by a vertical zipper. The inner chamber is surrounded by the outer chamber, formed by a roof, wings, trapezoid-cut walls, a longitudinal row of holes, and four walls also closed by a vertical zipper. This design avoids the direct biting of anthropophilic mosquitoes on the human individual that sits in the inner chamber. The four corners may have sticks passing inside sheaths built in the material (Figs 2 and 4) or strings that attach the trap to the gazebo-like tent. Mosquitoes are allowed to enter the trap through spaces located at the bottom of the outer chamber, lifted 10 cm from the ground.

**Fig 5:**
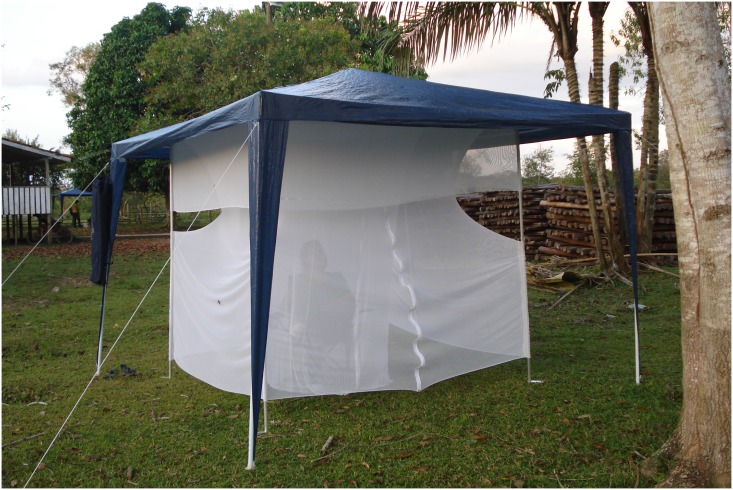
The MosqTent fully deployed using aluminium sticks inserted into sheaths sewn in the walls´ corners used with a commercially available gazebo-like tent (in blue).

The addition of a commercially available gazebo-like tent (Figs 3 and 5) allows the MosqTent to be used in low to moderate rain conditions.

### Ethical considerations

Mosquito collections were carried out under permit #1922008 of the Brazilian Institute for the Environment and Renewable Resources (Instituto Brasileiro do Meio Ambiente e dos Recursos Naturais Renováveis—IBAMA) and verbal authorization of the farm owner. The mosquito collections did not involve endangered or protected species and were not made in environmental-protected areas. The use of the HLC was approved by the Research Ethics Committee of the Institute of Scientific and Technological Research of the State of Amapá—IEPA (Official Letter 007/2008-CEP/IEPA). Anophelines were collected by experienced entomology technicians from IEPA or by the authors themselves. All technicians involved in the anthropophilic anopheline collections provided their written informed consent to participate in this study, were approved by the Research Ethics Committee of the Institute of Scientific and Technological Research of the State of Amapá, Brazil, and are registered in the IBAMA Federal Technical Register (Cadastro Técnico Federal—IBAMA).

## Results

### Comparison of results for black and white MosqTents, BG Sentinel, and HLC catches

#### Mosquito yields

During anopheline identification that occurred within 24 h after collection, it was visually verified that none of the mosquitoes collected by the MosqTent were engorged with blood. A total of 22,151 anophelines belonging to five species (including species complexes) that are all malaria vectors were caught over 20 nights for 4 h/night, repeating a 5 x 5 Latin square four times in different months to compare the MosqTent catch with a range of catching methods for sampling the five main species of anthropophilic anopheline species in Amapá, Brazil (Table 2).

**Table 2:** Number of anopheline vectors collected by the MosqTent tested against other anopheline collection methods in Cariobal, Amapá State, Brazil. Highest values are shaded.

Anopheline species/ Collection method	Month and year				Species subtotal (%)	(Total %)
*An*. *marajoara*	Aug 2009	Oct 2009	Jan 2010	Apr 2010		
HLC	532	836	313	426	2,107 (15.1)	
protected HLC	501	1318	266	469	2,554 (18.3)	
BG using CO_2_	569	459	318	340	1,686 (12.1)	
MosqTent white	875	1,271	839	1,048	4,033 (28.9)	
MosqTent black	858	1093	762	877	3,590 (25.7)	
**Subtotal (%)**	**3,335**	**4,977**	**2,498**	**3,160**	**13,970**	**(63.1)**
*An*. *triannulatus*						
HLC	689	325	120	126	1,260 (25.8)	
protected HLC	495	365	118	205	1,183 (24.3)	
BG using CO_2_	550	149	398	529	1,626 (33.4)	
MosqTent white	186	68	97	143	494 (10.1)	
MosqTent black	117	43	66	86	312 (6.4)	
**Subtotal (%)**	**2,037**	**950**	**799**	**1,089**	**4,875**	**(22)**
*An*. *darlingi*						
HLC	627	64	72	143	906 (35.5)	
protected HLC	484	125	65	110	784 (30.7)	
BG using CO_2_	119	12	0	31	162 (6.3)	
MosqTent white	199	46	34	41	320 (12.5)	
MosqTent black	233	47	22	78	380 (14.9)	
**Subtotal (%)**	**1,662**	**294**	**193**	**403**	**2,552**	**(11.5)**
*An*. *braziliensis*						
HLC	34	46	4	2	86 (17.6)	
protected HLC	37	90	6	0	133 (27.2)	
BG using CO_2_	37	2	0	0	39 (8)	
MosqTent white	45	29	13	12	99 (20.2)	
MosqTent black	39	68	12	13	132 (27.0)	
**Subtotal (%)**	**192**	**235**	**35**	**27**	**489**	**(2.2)**
*An*. *nuneztovari*						
HLC	9	10	20	2	41 (15.5)	
protected HLC	15	15	8	9	47 (17.7)	
BG using CO_2_	5	1	28	10	44 (16.6)	
MosqTent white	19	7	55	11	92 (34.7)	
MosqTent black	15	7	14	5	41 (15.5)	
**Subtotal (%)**	**63**	**40**	**125**	**37**	**265**	**(1.2)**
**TOTAL (%)**	**14,578**	**12,992**	**7,300**	**9,432**	**22,151**	**(100)**

Cariobal is a malaria-endemic area where the most prevalent anopheline species is the highly anthropophilic *An*. *marajoara*, with 63.1% of the total mosquitoes collected (or 13,970 specimens, Table 2).

In absolute numbers, the MosqTent collected *An*. *marajoara*, *An*. *braziliensis* Chagas, 1907, and *An*. *nuneztovari* Gabaldón, 1940, catching approximately 30% of the specimens for these three species (Table 2). For the anthropophilic *An*. *darlingi*, considered the main malaria vector in Brazil, the collection methods with highest yields were the HLC and the protected HLC (36% and 30%, respectively, Table 2). As expected from previous studies, BG-sentinel traps using CO_2_ as an attractant performed poorly for *An*. *darlingi*. The white and black MosqTents yielded half of the HLC and protected HLC yields for *An*. *darlingi* (Table 2).

**Fig 6:**
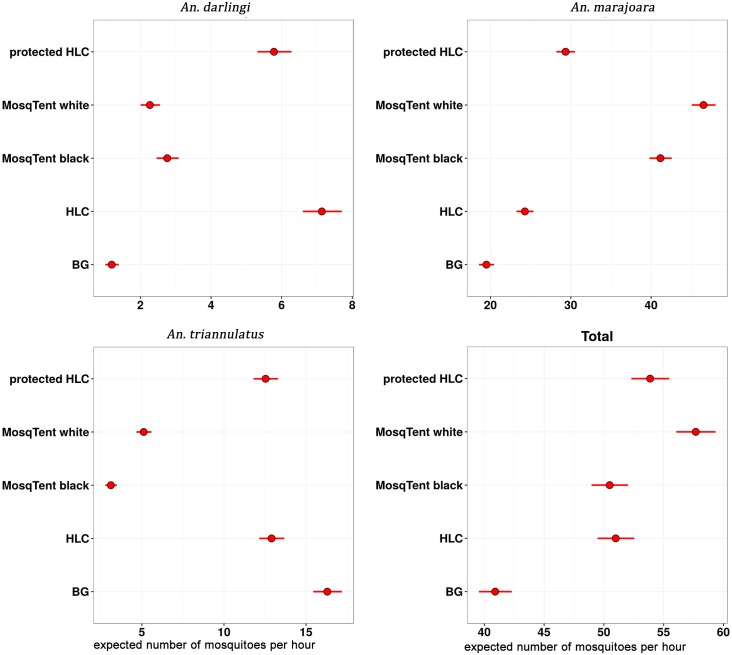
Posterior median (95% credible interval) of the expected number of mosquitoes per hour (*x*-axis) for *An*. *darlingi*, *An*. *marajoara*, *An*. *triannulatus*, and total anophelines per collection method (protected HLC, MosqTent white and black, HLC, and BG-sentinel traps).

Nonetheless, estimates of the posterior median and the 95% credible intervals of the collection rates for each collection method, controlling per collection point, month, day, and collection period showed that the HLC most efficiently collected *An*. *darlingi* and that the MosqTents (white and black) were the most efficient in collecting *An*. *marajoara* compared to the other trapping methods (Fig 6 and Table 3). On the other hand, the BG Sentinel was more efficient in collecting *An*. *triannulatus* than the other methods (Fig 6 and Table 3).

**Table 3:** Posterior median (95% credible interval) of the expected number of mosquitoes per hour by collection method and anopheline species (*An darlingi*, *An marajoara*, *An*. *triannulatus*, and total anophelines) in Cariobal, Macapá, Amapá State, Brazilian Amazon. Highest values are shaded.

Collection method	*An*. *darlingi*	*An*. *marajoara*	*An*. *triannulatus*	Total
HLC	7.1 (6.6, 7.7)	24.3 (23.2, 25.4)	12.9 (12.2, 13.7)	51.0 (49.5, 52.5)
BG	1.2 (1.0, 1.4)	19.5 (18.6, 20.5)	16.3 (15.4, 17.2)	40.9 (39.6, 42.3)
MosqTent black	2.8 (2.5, 3.1)	41.2 (39.8, 42.6)	3.1 (2.8, 3.5)	50.5 (49.0, 52.0)
MosqTent white	2.3 (2.0, 2.6)	46.5 (45.1, 48.0)	5.1 (4.7, 5.6)	57.7 (56.1, 59.3)
Protected HLC	5.8 (5.3, 6.3)	29.3 (28.2, 30.5)	12.5 (11.8, 13.3)	53.9 (52.3, 55.5)

**Fig 7:**
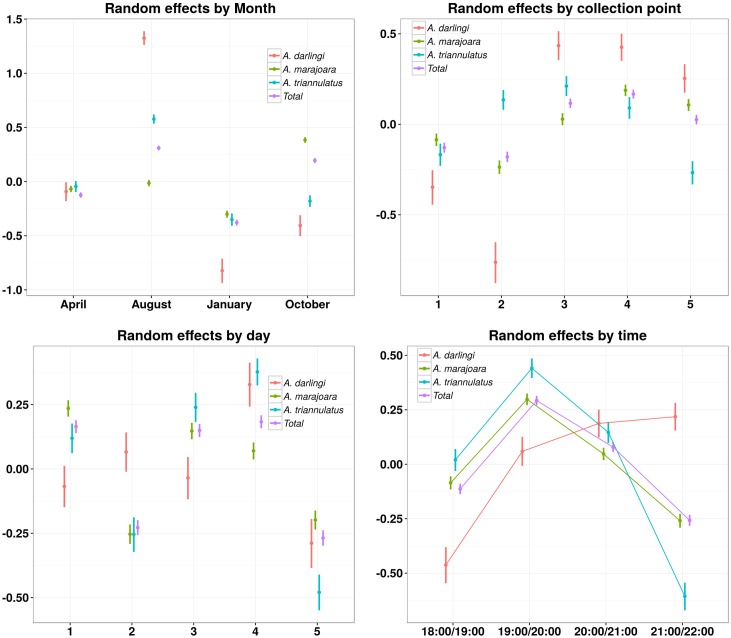
Random effects for the multilevel poisson model for the number of *An*. *darlingi*, *An*. *marajoara*, *An*. *triannulatus*, and total anophelines collected by method, controlled by month, collection point, day, and time. Each random effect is the contribution to the logarithm of the expected number of mosquitoes. The further from zero (*y*-axis), the greater the effect of the variable in the mosquito yield.

For all mosquito species, the collection method is the most important effect in order to explain the variability of mosquito capture rates (Table 4). Data suggests that for *An*. *darlingi*, the month of collection also played an important role (Table 4 and Fig 7).

**Table 4:** Posterior mean of the precision parameter for the random effects of collection method, month, collection point, day, and time (hour of the day) for *An*. *darlingi*, *An*. *marajoara*, *An*. *triannulatus*, and total anophelines in Cariobal, Macapá, Amapá State, Brazilian Amazon. The lower the precision the more important is the effect to explain data variability (shaded).

Precision	*An*. *darlingi*	*An*. *marajoara*	*An*. *triannulatus*	Total
Collection method	0.80	0.12	0.28	0.09
Month	1.88	21.41	10.00	17.88
Collection point	5.22	54.22	34.59	65.83
Day	11.29	12.53	5.21	11.89
Time	17.14	17.63	5.92	16.72

Estimated controlled random effects show that month and hour of collection seem more relevant for the three species (Fig 7). *An*. *darlingi* and *An*. *triannulatus* were likely to be observed mostly in August, while *An*. *marajoara* was likely to be observed in October. All the species were observed mainly at dusk, starting from 1800h until 2000h, while *An*. *darlingi* was seen later, at 2100h–2200h.

### Parity

A total of 1,385 *An*. *darlingi* and 5,618 *An*. *marajoara* females had their ovaries dissected to determine parity status. No differences in the abundance of parous mosquitoes among traps was observed between *An*. *darlingi* (*X*^*2*^ = 3.55, df = 4, *p* = 0.47, Table 5) and *An*. *marajoara* (*X*^*2*^ = 3.03, df = 4, *p* = 0.55, Table 5).

**Table 5:** Number of parous *An*. *darlingi* and *An*. *marajoara* females collected in the MosqTent against other collection methods in Cariobal, Macapá, Amapá State, the Brazilian Amazon.

Type of Trap/Anopheline	Human landing catch	Protected human landing catch	BG-sentinel + CO2	MosqTent white	MosqTent black
*An*. *darlingi*					
Parous	263	248	44	112	151
(%)	(58.3)	(58.4)	(48.4)	(59.6)	(65.7)
**Total**	**451**	**425**	**91**	**188**	**230**
*An*. *marajoara*					
Parous	488	544	416	694	706
(%)	(50.4)	(53.9)	(49.3)	(50.8)	(49.3)
**Total**	**969**	**1,008**	**844**	**1,365**	**1,432**

## Discussion

In an ever-changing environment in which mosquito vector species distributions vary continuously, due not only to distinct regional differences [23] but also due to ecological changes and adaptation [24], mosquito vector collections are routinely needed. All traps from the least-demanding anthropophilic mosquito collection methods, such as, CDC light traps, BG Sentinel, and Mosquito Magnet traps, to more labor-intensive methods, such as Shannon-type tents, use human-produced molecules, from simple to complex, to mimic human attraction and, hence, mosquito yields [4,5,6,7,8,9,10].

For anthropophilic mosquitoes, there has been no better and more productive way of collection than the use of a human individual as an attractant as in the HLC [12,25–28]. The use of the HLC as a gold standard for the assessment of anthropophilic mosquitoes will continue to be used until a trap that can lure and collect as many mosquitoes as efficiently as the HLC is available.

The MosqTent was field tested in an area with highly anthropophilic malaria vectors in the Brazilian Amazon against other collection methods that were previously tested as yielding high numbers of Brazilian malaria vectors, (BG-sentinel + CO_2_ and protected HLC) [10,16]. During MosqTent testing, the clockwise rotation may have led to neighboring effect since this rotation did not follow a complete randomized collection point change. Nevertheless, this effect might have been reduced by the 50 m distance guarded among collection methods. The HLC and the protected HLC had statistically similar yields.

Parity is a proxy to mosquito age and gonotrophic cycle and, therefore, a major entomological parameter in the epidemiology of mosquito vector–borne diseases. A suitable mosquito sampling method should provide an accurate figure of this parameter. The MosqTent collected the two most important malaria vectors in the area, *An*. *darlingi* and *An*. *marajoara*, with the same parity proportions as the HLC (58.3% for HLC and 59.6% for MosqTent white for *An*. *darlingi*, and 50.4% for HLC and 50.8% for MosqTent white for *An*. *marajoara*, Table 5). The same species caught by HLC were also collected by the MosqTent (Table 2). Regarding yield, for *An*. *marajoara*, nearly twice as many specimens were caught by both tent traps than the HLC (25.7% MosqTent white and 28.9% MosqTent black compared to 15.1% for HLC, Table 2). *An*. *marajoara* is an anthropophilic species belonging to the Albitarsis complex. In Cariobal, *An*. *marajoara* was present in high density (63% or 13,970 specimens out of the total of 22,151 specimens, Table 2). The high efficiency of the MosqTent is probably due to the high number of *An*. *marajoara* specimens. In high mosquito–density conditions, during the HLC collection, many mosquitoes bite and escape since the collector is heavily attacked and cannot collect efficiently. On a previous occasion [29], the MosqTent was able to collect an average of 3,000 anophelines/man-hour compared to only 240 anophelines/man-hour for the HLC (Table 2). In these high mosquito–density conditions, field workers are more prone to get infected in endemic malaria areas. Possibly, this is one of the best qualities of the MosqTent, i.e., the collection of a high number of specimens of anthropophilic mosquito species in high-density conditions. For *An*. *triannulatus*, BG was as good as HLC, and tent traps were much lower (33.4% BG when compared to 10.1% MosqTent white and 6.4% MosqTent black, Table 2). All *An*. *triannulatus* species members of the Triannulatus complex are considered zoophilic [30]. Thus, the low efficiency in collecting *An*. *triannulatus* might be due to the human collector present in the inner chamber of the MosqTent, which better attracts anthropophilic mosquito species. For *An*. *darlingi* in Cariobal, both tent traps caught half the amount of the HLC (14.9% for MosqTent black and 12.5% for MosqTent white while HLC collected 35.5%, Table 2). Conceivably, the MosqTent may have a minimum threshold to efficiently collect. *An*. *darlingi* in Cariobal was present in low density (2,552 specimens or 11.5% out of the 22,151 total specimens, Table 2). In this condition, when an anthropophilic mosquito species is present in low density, the MosqTent might not be able to determine which species are present as accurately as the HLC. For *An*. *braziliensis*, there was no significant difference between the MosqTent white (20.2%) and the HLC (17.6%, Table 2). *An*. *braziliensis* was present in very low density in Cariobal, even less than *An*. *darlingi* (489 specimens or 2.2% out of the 22,151 total specimens, Table 2). Nonetheless, contrary to the anthropophilic *An*. *darlingi*, also present in low density, the zoophilic *An*. *braziliensis* could be efficiently collected by the MosqTent white for reasons that cannot be explored by the current experiment design. Interestingly, *An*. *braziliensis* was caught in significantly high numbers in the protected HLC (27.2%) when the collector uses a thick black sock (Fig 1) and in the MosqTent black (27%, Table 2). Maybe for *An*. *braziliensis* the black color acts as an additional attractant. For *An*. *nuneztovari*, the MosqTent white collected twice as much as the HLC (34.7% MosqTent white and 15.5% for HLC, Table 2), while the MosqTent black collected the same proportions as the HLC (15.5%, Table 2). *An*. *nuneztovari* is a complex of cryptic species and was the species with the lowest density in Cariobal (265 specimens or 1.2% out of the 22,151 total specimens, Table 2). The efficiency in *An*. *nuneztovari* collection by the MosqTent white might indicate that an anthropophilic member of the complex is present in Cariobal. Besides anthropophily and population density, other dynamic biological and ecological factors might influence the collection efficiency of the MosqTent.

**Fig 8:**
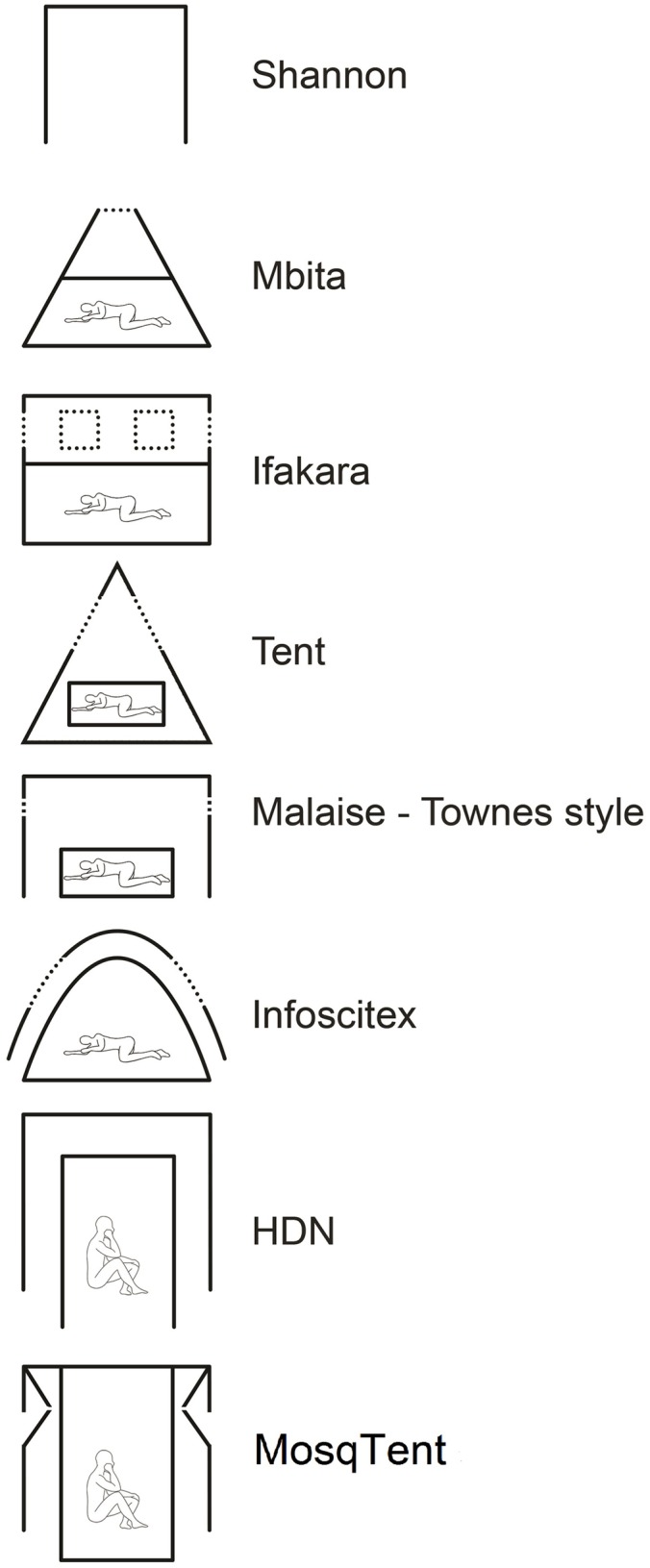
Simplified schemes of traps aiming to collect anthropophilic mosquitoes to show differences in their designs: Shannon (single chamber) [31], Mbita [32], Ifakara [33,34], Tent [35], Malaise (Townes style) [35], Infoscitex [36], Human Double Net-HDN [37], and MosqTent (this paper).

The main innovation characteristics of the MosqTent are its design (side wings that increase the collection efficiency) and portability (~1 kg bag that opens up in a full trap in less than 2 min, S1 Supporting information) when compared to other double-chamber mosquito traps (Fig 8). The side wings were added after the observation that when mosquitoes come avid to feed, they encounter the outer net wall. The mosquitoes then start hopping towards the upper side of the wall and are conducted by the wings to the outer chamber from where they try to pass through the second net wall, but, failing to bite, they also cannot leave the outer chamber (red dotted lines in Fig 4B).

Given the promising data that has resulted from our studies, the MosqTent’s efficiency should be tested in a range of other contexts in which medically important mosquitoes are not easily captured using the prevailing trapping techniques, and for a variety of other important mosquito vector species, whenever adult collection is required.

Further developments may include testing for other fabric colors, different mesh sizes and dimensions for other hematophagous insects and conditions, additional chemical mosquito attractants, and even the replacement of the human attractant for other attractants. MosqTent modifications that would allow the trap to be applied as a vector control tool with killing action could also be explored.

## Conclusions

The MosqTent is a newly developed, protective, double-chamber, portable, individual mosquito trap for anthropophilic mosquitoes that protects the collector from mosquito biting since the collector sits inside an inner chamber netted compartment. The same individual can serve as an attractant (in the inner chamber) and leave the inner chamber well dressed and protected to collect the mosquitoes trapped in the outer chamber after the luring period. The MosqTent aims to be used for research and entomological surveillance by field personnel. The MosqTent collected more mosquitoes than the HLC in high mosquito–density conditions. The MosqTent performed well by collecting a high number of specimens of *An*. *marajoara*, a local vector and anthropophilic mosquito species present in high density, but not so well in collecting *An*. *darlingi*, an anthropophilic mosquito species considered the main vector in Brazil but present in low-density conditions in the area. The HLC showed a higher efficiency in collecting *An*. *darlingi* in these low-density conditions. The MosqTent was able to catch comparable portions of parous females for both *An*. *darlingi* and *An*. *marajora* as the HLC. The results encourage further development and the use of this mosquito trap for anthropophilic vectors elsewhere.

## Supporting information

S1 Supporting Information Setting up of a MosqTent. (MP4)
