# Correction: MosqTent: An individual portable protective double-chamber mosquito trap for anthropophilic mosquitoes

**DOI:** 10.1371/journal.pntd.0005578

**Published:** 2017-05-02

**Authors:** The PLOS Neglected Tropical Diseases Staff

There is an error in reference 13. The correct reference is: WHO: Manual de entomologia prática aplicada à malaria, Pt. II, 1975.

A reference is omitted from the reference list. Reference 14 should be: World Health Organization. International Ethical Guidelines for Epidemiological Studies. Biomedical Research 1–113. Geneva; World Health Organization, 2008.

There is an error in reference 31. The correct reference is: Mathenge EM, Killeen GF, Oulo DO, Irungu LW, Ndegwa PN, Knols BG. Development of an exposure-free bednet trap for sampling Afrotropical malaria vectors. Med Vet Entomol. 2002; 16(1): 67–74.

Reference 32 is present in error and should not be included in the reference list.

As a result of these errors, the reference list is out of order. Reference 14 in the list should be reference 15, reference 15 should be reference 16, reference 16 should be reference 17, and so on, as far as reference 31, which should be reference 32.
